# Inadequate prenatal care utilization and associated factors in São Luís, Brazil

**DOI:** 10.1186/1471-2393-14-266

**Published:** 2014-08-10

**Authors:** Ariane Cristina Ferreira Bernardes, Raimundo Antonio da Silva, Liberata Campos Coimbra, Maria Teresa Seabra Soares de Britto Alves, Rejane Christine de Sousa Queiroz, Rosângela Fernandes Lucena Batista, Heloisa Bettiol, Marco Antônio Barbieri, Antônio Augusto Moura da Silva

**Affiliations:** Department of Public Health, Federal University of Maranhão, Brazil, Rua Barão de Itapary, 155, Centro, São Luís, Maranhão 65020-070 Brazil; Department of Nursing, Federal University of Maranhão, Brazil, Rua Viana Vaz, 230, Centro, São Luís, Maranhão 65020-660 Brazil; Department of Puericulture and Pediatrics, Faculty of Medicine of Ribeirão Preto, University of São Paulo, Ribeirão Preto, Brazil, Avenida Bandeirantes, 3900, Hospital das Clínicas de Ribeirão Preto, Monte Alegre, Ribeirão Preto, São Paulo 14049-900 Brazil

**Keywords:** Prenatal care, Maternal welfare, Delivery of health care, Health evaluation, Health status disparities, Antenatal care

## Abstract

**Background:**

Over the last decades there has been a reduction of social inequalities in Brazil, as well as a strong expansion of health services, including prenatal care. The objective of the present study was to estimate the rate of inadequate prenatal care utilization and its associated factors in São Luís, Brazil, in 2010 and to determine whether there was a reduction of inequity in prenatal care use by comparing the present data to those obtained from a previous cohort started in 1997/98.

**Methods:**

Data from the BRISA (Brazilian birth cohort studies of Ribeirão Preto and São Luís) population-based cohort, which started in 2010 (5067 women), were used. The outcome variable was the inadequate utilization of prenatal care, classified according to the recommendations of the Brazilian Ministry of Health. The explanatory variables were organized into three hierarchical levels based on the Andersen’s behavioral model of the use of health services: predisposing, enabling and need factors.

**Results:**

Only 2.0% of the women did not attend at least one prenatal care visit. The rate of inadequate prenatal care utilization was 36.7%. Despite an improved adequacy of prenatal care use from 47.3% in 1997/98 to 58.2% in 2010, social inequality persisted: both low maternal schooling (prevalence ratio (PR) = 2.78; 95% confidence interval (95% CI) 2.23-3.47 for 0 to 4 years of study) and low family income, less than 0.5 monthly minimum wage per capita (PR = 1.37; 95% CI 1.22-1. 54), continued to be associated with higher rates of inadequate prenatal care utilization. Racial disparity regarding adequate utilization of prenatal services was detected, with black (PR = 1.19; 95% CI 1.04-1.36) and mulatto (PR = 1.14; 95% CI 1.02-1.26) women showing higher rates of inadequate use. On the other hand, women covered by the FHP - Family Health Program (PR = 0.92; 95% CI 0.85-0.98) showed a lower rate of inadequate prenatal care utilization.

**Conclusions:**

Despite strong expansion of health services and expressive improvements in adequate prenatal care use and social indicators, inequalities in prenatal care use still persist. The FHP seems to be effective in reducing inadequate prenatal care utilization.

## Background

Appropriate care during pregnancy and childbirth is essential to reduce the magnitude of mother-child morbidity and mortality. The estimate is that one-quarter of infant deaths and almost all maternal deaths are due to inadequate delivery of health care from the beginning of pregnancy to the immediate postpartum period [1]. Mother-child morbidity and mortality are quite elevated in Brazil compared to developed countries [1, 2]. Although much has been done to expand the delivery of mother-child health care in Brazil, situations of insufficient facilities, low-quality services and social disparity in access still persist [3–5].

Since the 1970 decade, several studies evaluating prenatal care use have suggested indices for its classification. Particularly important among them are the Kessner index [6], proposed in 1973, and the Kotelchuck index [7], proposed in 1994 and known as Adequacy of Prenatal Care Utilization (APNCU) index. All of these indices are based on the minimum number of visits recommended by the American College of Obstetricians and Gynecologists (ACOG), which ranges from 11 to 14 visits. In contrast, the Brazilian Ministry of Health recommends at least six visits for a low-risk term pregnancy with an early beginning of prenatal care, up to the fourth month of pregnancy [8–11] whereas the World Health Organization (WHO) recommends a minimum of four prenatal care visits [12].

Some theoretical models have been developed to explain the use of health services, such as the Andersen’s behavioral model [13], the health belief model [14], the Dutton’s model [15], and the Evans & Stoddart’s model [16]. Among them, the Andersen’s model [13], consisting of predisposing characteristics, enabling resources and need, has been the one most frequently applied both in utilization and access studies.

Some studies [4, 8, 17, 18] have pointed out that a significant number of women continue to receive inadequate prenatal care, especially women of low educational level, younger than 20 years, of low family income, of mulatto and black ethnicity, multiparous, smokers, living without a companion, and women covered by public health services. It has been demonstrated that disparities in socioeconomic, demographic and behavioral factors continue to be important characteristics associated with inadequate prenatal care use in developing countries, indicating persistence of social inequity [3, 9, 10, 19–22].

In the state of Maranhão, Northeastern Brazil, prenatal care coverage (proportion of women having attended prenatal care at least once) was 71.3% in 1996 [23], increasing to 85.6% in 2007/2008 [24]. However, when considering adequate prenatal care coverage (as recommended by the Ministry of Health), this value was much lower, 43.4%, in 2007/2008 [24].

Despite all the efforts of the Brazilian Ministry of Health in partnership with States and Municipalities to expand the coverage of prenatal care, the North/Northeast regions have worse prenatal care indicators regarding the number of visits and the time when they are started, revealing important regional differences [25].

A reduction of social disparities, due to increases in income associated with economic development and to a conditional cash transfer program [26], and a strong expansion of health services, including prenatal care and the family health program (FHP) [27], have occurred in Brazil over the last decades.

Thus, the objective of the present study was to determine the rate of inadequate prenatal care utilization and its associated factors in the municipality of São Luís, Maranhão, Brazil in 2010. We also determined whether there was a reduction of disparities in inadequate prenatal care use by comparing the present data to those obtained for a previous cohort started 13 years ago in 1997/98.

## Methods

This was a cross-sectional study nested in the BRISA cohort (Brazilian Birth Cohort Studies of Ribeirão Preto and São Luís). The data for the present study were obtained from hospital singleton live births of the São Luís birth cohort, started in 2010.

The population-based cohort was formed throughout the year of 2010, including births at ten public, private or insurance-covered hospitals and maternities, which provided delivery and neonatal care. At the time of the study, 98% of all births occurred in hospitals. We did not include maternities performing less than 100 deliveries per year, which corresponded to 3.3% of the births. Thus, the target population consisted of 94.7% of all births.

The sample was stratified by maternity hospital with the same sampling fraction in each hospital. In each maternity hospital sampling was systematic and all live births and stillbirths were listed in order of occurrence. The sampling interval was three. A random number from 1 to 3 was drawn to determine the starting point for each study unit. Thus one out of three births were randomly selected for interview. A total of 5451 women having given birth and residing in the municipality for at least 3 months were invited to participate. There was a 4.6% loss due to refusal or early discharge, resulting in a sample of 5212 women. After the exclusion of 99 multiple deliveries and 70 stillbirths, the final sample of the present study consisted of 5067 births.

The minimum sample size was fixed at 5000 births. This sample size allowed estimation of 50% prevalences (maximum p × q product) with 2% precision and 99% confidence level. It was also possible to compare two proportions, considering a 5% probability of type I error, 80% study power, working with the maximum p × q product (a 50% prevalence of the event) and fixing at 4% the minimum significant difference. For prevalences of less than 50% it was possible to detect lower differences.

A standardized questionnaire was applied to the women having given birth for data collection, preferably during the first 24 hours after delivery. Information about prenatal care was obtained from the mothers’ verbal reports and from their charts, when available.

The use of prenatal care was considered adequate when it was started up to the 4^th^ month of pregnancy, with a minimum of six visits for a term pregnancy or a smaller number according to gestational age, i.e., at least five visits for a pregnancy of 33-36 weeks, four visits for a pregnancy of 29 to 32 weeks, and two visits for a pregnancy concluded after less than 24 weeks of gestational age. All other situations were considered inadequate. The women who did not use prenatal care or were unable to inform about the number of visits or the trimester when prenatal care had begun were assigned to the “missing” category [8].

Gestational age was calculated from the date of the last menstrual period reported by the mother. The 15^th^ day of the month was imputed when the mother did not know the exact day, but only remembered the month of the last menstruation (416 cases). The day or month of the last menstrual period was not available in 272 interviews, corresponding to 5.4% of the total sample. Cases of implausible gestational age (less than 20 or more than 43 weeks) were recoded as missing (174). Cases located above the 99th percentile of the English reference curve [28] in which the date of the last menstrual period was incompatible with birth weight were also reclassified as missing (96 cases). At the end, gestational age was imputed in a regression model based on birth weight, parity, per capita monthly family income, and newborn’s sex. A total of 446 cases were imputed, 39 of them as preterm births and 407 as term births.

The outcome was inadequate prenatal care use. The explanatory variables were defined considering three hierarchized levels according to Andersen’s behavioral model [13] for the use of prenatal care services: predisposing characteristics – marital status (married, consensual union, with no companion), maternal schooling as full years of study (0 to 4, 5 to 8, 9 to 11, ≥12), maternal age in years (<20, 20 to 34, ≥35 years), parity (1, 2 to 4, ≥5 children), mother’s self-reported skin color (white, black or mulatto) and whether the mother was the family head (yes or no); enabling resources – monthly per capita family income as number of minimum wages (≤0.5, 0.5 |- 1, ≥1, missing), category of prenatal care (public when the mother was covered by the Unified Health System, or private when the visits were covered by insurance/health plan or by a private office) and coverage by the Family Health Program, which includes a home visit by the community health worker and visits to doctors/nurses at a health facility, classified as yes or no; need factors – previous pregnancy loss (a history of miscarriage or stillbirths, classified as yes or no); previous preterm birth (yes or no); arterial hypertension before or during pregnancy (yes or no), maternal smoking during pregnancy (yes, when the mother smoked at least one cigarette per day during pregnancy, or no), and maternal consumption of alcohol during pregnancy (yes, when the mother had ingested beer, wine, whiskey, vodka, gin or rum during pregnancy, or no otherwise).

Data were analyzed by descriptive statistics (frequencies and percentages), and the proportions were compared by the chi-square test.

Bivariate analysis was first performed, with the estimate of non-adjusted prevalence ratios (PR) and 95% confidence interval (95% CI). Poisson regression analysis with robust variance adjustment was performed to identify factors associated with inadequate prenatal care utilization in order to attenuate a possible underestimate of the standard error, considering that the dependent variable is binary and that its prevalence was higher than 10%. The level of significance was set at 0.05.

All independent variables were used in multivariable analysis. Hierarchized modeling was used, with adjustment being first performed for the predisposing characteristics in the first block. Adjustment for predisposing characteristics and enabling resources was performed in the second block, and adjustment for all variables (predisposing, enabling, and need factors) was performed in the third block. The significance of the association of each variable was determined in the block to which the variable was entered first. Based on Andersen’s theoretical model of health services utilization [13] all variables were kept in the models, regardless of whether they improved the model's goodness of fit. In bivariate and multivariable analysis, 260 cases were excluded because of lack of information about the number of visits and/or the trimester when prenatal care was started. All analyses were carried out with the aid of the Stata 12 software.

The Research Ethics Committee of the University Hospital President Dutra (protocol no. 4771/2008-30) approved the present study. Written informed consent to participate in the study was obtained from all participants.

## Results

Table 1 presents the distribution of characteristics of prenatal care utilization in São Luís in 2010. Prenatal care coverage, measured on the basis of at least one visit at any time during pregnancy, was 98.0%. Six or more prenatal visits were attended by 60.5% and 66.2% started prenatal care during the first trimester of pregnancy. Eighty percent of the visits occurred in the public sector of the Unified Health System (SUS in the Portuguese acronym). The rate of inadequate prenatal care use was 36.7%, reaching 38.7% when cases without information about the number of visits or the time when prenatal care was started were excluded.

**Table 1:** **Characteristics of prenatal care use based on the minimum calendar of the ministry of health**

Variables	n	%
**Received prenatal care**		
Yes	4,968	98.0
No	99	2.0
**Number of visits**		
None	99	2.0
1 – 3	521	10.3
4 – 5	1,122	22.1
≥ 6	3,065	60.5
Missing	260	5.1
**Starting trimester**		
No prenatal care	99	2.0
1st trimester	3,356	66.2
2nd trimester	1,395	27.5
3rd trimester	117	2.3
Missing	100	2.0
**Site of prenatal care**		
No prenatal care	99	2.0
Public sector of SUS	4,052	80.0
Private	218	4.3
Insurance/Health plan	690	13.6
Missing	8	0.1
**Classification of prenatal care**		
Adequate*	2,949	58.2
Inadequate	1,858	36.7
Missing	260	5.1
Total	5067	100

São Luís, Brazil, 2010.

*Prenatal care was considered to be adequate when started up to the fourth month of pregnancy and when it involved at least six visits for a term pregnancy or a smaller number of visits according to gestational age. The missing category corresponds to 260 cases without information about the number of visits and/or the trimester when prenatal care was started (source: ref. 10). SUS: Unified Health System in the Brazilian acronym.

Table 2 describes the socioeconomic, demographic, reproductive, behavioral and morbidity characteristics of the women having given birth. The percentage of adolescent mothers was 18.7%, the percentage of women aged ≥35 years was 7.6%, and 47.6% were primiparae. Few women (4.6%) had low schooling (0 to 4 years). Most women lived in consensual union (59.2%) and were mulatto (68.5%). Few women were family heads (9.3%). A low monthly per capita family income (<0.5 minimum wage) was observed in 18.3%, 81.7% received prenatal care in public facilities and the Family Health Program covered 31.7%. Hypertension was reported by 17.9%, diabetes by 2.3%, a previous pregnancy loss by 23.2%, and a previous preterm birth by 14.4%. Few women smoked during pregnancy (4.0%) and 14.5% consumed alcohol.

**Table 2:** **Socioeconomic, demographic, reproductive, behavioral, and morbidity characteristics of women having given birth**

Variables	N	%
**Predisposing characteristics**		
**Maternal age (years)**		
< 20	945	18.7
20 – 34	3,733	73.7
≥ 35	389	7.6
**Parity**		
1	2,412	47.6
2 – 4	2,480	48.9
≥ 5	175	3.5
Maternal schooling (years)*		
0 – 4	230	4.6
5 – 8	1,129	22.3
9 – 11	2,935	58.1
≥ 12	759	15.0
**Marital status**		
No companion	964	19.0
Married	1,106	21.8
Consensual union	2,997	59.2
Mother’s skin color*		
White	932	18.7
Black	644	12.8
Mulatto	3,419	68.5
**Family head**		
Mother	470	9.3
Others	4,597	90.7
**Enabling resources**		
**Monthly per capita family income in minimum wages**		
≥1	1,804	35.6
≥0.5 to <1	1,424	28.1
<0.5	929	18.3
Missing	910	18.0
Category of prenatal care *		
Private	908	18.3
Public	4,052	81.7
**Coverage by the family health program**		
Yes	1,606	31.7
No	3,461	68.3
**Need**		
Hypertension before and during pregnancy*		
Yes	908	17.9
No	4,158	82.1
Diabetes before or during pregnancy*		
Yes	115	2.3
No	4,948	97.7
**Previous pregnancy loss**		
Yes	1,177	23.2
No	3,890	76.8
**Previous preterm birth**		
Yes	726	14.4
No	4,321	85.6
**Maternal smoking during pregnancy**		
Yes	204	4.0
No	4,863	96.0
**Alcohol consumption during pregnancy**		
Yes	734	14.5
No	4.333	85.5

São Luís, Brazil, 2010.

*Totals for these variables vary because of missing values.

In non-adjusted analysis (Table 3), all predisposing and enabling factors were significantly associated with inadequate prenatal care utilization (p < 0.05). Regarding the need block, only previous pregnancy loss and previous preterm birth were not significantly associated with a higher rate of inadequate prenatal care use.

**Table 3:** **Non-adjusted analysis of the factors associated with inadequate prenatal care utilization**

Variables	n	Inadequacy (%)	PR *	(95% CI)	p-value
**Predisposing characteristics**					
**Maternal age (years)**					<0,001
< 20	878	50.6	1.35	1.25-1.46	
20 – 34	3,551	37.3	1		
≥ 35	378	23.3	0.62	0.52-0.75	
**Parity**					<0.001
1	2,297	31.9	0.74	0.68-0.79	
2 – 4	2,342	43.3	1		
≥ 5	168	66.1	1.53	1.36-1.72	
**Maternal schooling (years)**					<0.001
0 – 4	209	64.6	4.55	3.71-5.58	
5 – 8	1,066	56.8	4.00	3.32-4.81	
9 – 11	2,782	36.3	2.56	2.13-3.07	
≥ 12	740	14.2	1		
**Marital status**					<0.001
With no companion	906	50.1	2.38	2.08-2.71	
Married	1,067	21.1	1		
Consensual union	2,834	41.6	1.97	1.74-2.23	
**Mother’s skin color**					<0.001
White	883	28.5	1		
Black	606	41.9	1.47	1.28-1.69	
Mulatto	3,250	40.9	1.43	1.28-1.60	
**Family head**					<0.001
Mother	454	27.8	1		
Others	4,353	39.8	1.43	1.23-1.67	
**Enabling resources**					
**Monthly per capita family income in minimum wages**					<0.001
≥1	1,750	22.5	1		
≥0.5 to <1	1,369	43.8	1.94	1.75-2.16	
<0.5	858	54.5	2.42	2.18-2.70	
Missing	830	47.8	2.12	1.90-2.38	
**Category of prenatal care**					<0.001
Private	875	10.1	1		
Public	3,826	43.6	4.34	3.55-5.31	
**Coverage by the family health program**					0.019
No	3,300	37.6	1		
Yes	1,507	41.1	1.09	1.02-1.18	
**Need**					
**Hypertension before or during pregnancy**					<0.001
Yes	873	33.3	0.84	0.76-0.93	
No	3,933	39.8	1		
**Diabetes before or during pregnancy**					<0.001
Yes	114	20.2	0.52	0.36-0.75	
No	4,689	39.0	1		
**Previous pregnancy loss**					0.092
Yes	1,116	36.5	0.93	0.85-1.01	
No	3,691	39.3	1		
**Maternal smoking during pregnancy**					<0.001
Yes	189	69.8	1.87	1.69-2.07	
No	4,618	37.4	1		
**Alcohol consumption during pregnancy**					<0.001
Yes	679	49.8	1.35	1.24-1.48	
No	4,128	36.8	1		
**Previous preterm birth**					0.171
Yes	691	41.0	1.07	0.97-1.18	
No	4,096	38.3	1		

São Luís, Brazil, 2010.

PR – Prevalence ratio; CI – Confidence interval.

*Prevalence ratio (PR) estimated by the Poisson regression model with robust adjustment of the standard errors. Totals for these variables vary because of missing values. A total of 210 cases were excluded because of lack of information about the number of visits and/or the trimester prenatal care was started.

In the adjusted hierarchized model, the following factors were associated with lower rates of inadequate prenatal care use in the block of predisposing characteristics (Table 4): age ≥35 years (PR = 0.64; 95% CI 0.54-0.78) and primiparity (PR = 0.70; 95% CI 0.64-0.77). Higher rates of inadequate prenatal care use were observed for women aged less than 20 years (PR = 1.28; 95% CI 1.17-1.40), who gave birth to five or more children (PR = 1.31; 95% CI 1.16-1.49) and who were not the head of family (PR = 1.17; 95% CI 1.01-1.35). The rates of inadequate prenatal care use increased with decreasing maternal schooling, and the highest rate of inadequacy was observed in the category of zero to four years of study (PR = 2.78; 95% CI 2.23-3.47). Having no companion (PR = 1.41; 95% CI 1.25-1.60) and living in consensual union (PR = 1.81; 95% CI 1.58-2.10) were associated with higher rates of inadequacy. Women who self-reported brown (PR = 1.14; 95% CI 1.02-1.26) or black (PR = 1.19; 95% CI 1.04-1.36) skin colors also had higher rates of inadequate prenatal care use.

**Table 4:** **Adjusted analysis by means of hierarchized modeling of the factors associated with inadequate prenatal care use**

	Block 1		Block 2		Block 3	
Variables	PR (95% CI)*	p-value	PR (CI 95%)*	p-value	PR (95% CI)*	p-value
**Predisposing characteristics**						
**Maternal age (years)**		<0.001				
< 20	1.28 (1.17 - 1.40)					
20 – 34	1.00					
≥ 35	0.64 (0.54 - 0.78)					
**Parity**		<0.001				
1	0.70 (0.64 - 0.77)					
2 – 4	1.00					
≥ 5	1.31 (1.16 - 1.49)					
**Maternal schooling (years)**		<0.001				
0 – 4	2.78 (2.23 - 3.47)					
5 – 8	2.49 (2.04 - 3.05)					
9 – 11	1.86 (1.54 - 2.25)					
≥ 12	1.00					
**Marital status**		<0.001				
With no companion	1.81 (1.58 - 2.10)					
Married	1.00					
Consensual union	1.41 (1.25 - 1.60)					
**Mother’s skin color**		0.021				
White	1.00					
Black	1.19 (1.04 - 1.36)					
Mulatto	1.14 (1.02 - 1.26)					
**Family head**		0.031				
Mother	1.00					
Others	1.17 (1.01 - 1.35)					
**Enabling resources**						
**Per capita family income**				<0.001		
≥1			1.00			
≥0.5 to <1	-		1.29 (1.15 - 1.44)			
<0.5	-		1.37 (1.22 - 1.54)			
Missing	-		1.32 (1.18 - 1.49)			
**Category of prenatal care**				<0.001		
Private	-		1.00			
Public	-		2.74 (2.18 - 3.43)			
**Coverage by the family health program**				0.014		
No	-		1.00			
Yes	-		0.92 (0.85 - 0.98)			
**Need**						
**Hypertension before or during pregnancy**						0.013
Yes	-		-		0.89 (0.80 - 0.98)	
No	-		-		1.00	
**Diabetes before or during pregnancy**						0.062
Yes	-		-		0.72 (0.51 - 1.01)	
No	-		-		1.00	
**Previous pregnancy loss**						0.002
Yes	-				0.87 (0.80 - 0.95)	
No					1.00	
**Maternal smoking during pregnancy**						<0.001
Yes	-		-		1.28 (1.13 -1.45)	
No	-		-		1.00	
**Alcohol consumption during pregnancy**						0.002
Yes	-		-		1.15 (1.05 – 1.26)	
No	-		-		1.00	
**Previous preterm birth**						0.390
Yes	-		-		1.04 (0.94 – 1.15)	
No	-		-		1.00	

São Luís, Brazil, 2010.

PR – Prevalence ratio; CI – Confidence interval;

*Prevalence ratio (PR) estimated by the Poisson regression model with robust adjustment of the standard errors. A total of 210 cases were excluded because of lack of information about the number of visits and/or the trimester prenatal care was started. Block 1 – adjusted for predisposing characteristics; Block 2 – adjusted for predisposing characteristics and enabling resources; Block 3 – adjusted for all variables – predisposing characteristics, enabling resources and need.

In the block of enabling resources, having a monthly per capita family income of ½ to <1 minimum wage (PR = 1.29; 95% CI 1.15-1.44), of less than ½ minimum wage (PR = 1.37; 95% CI 1.22-1.54), or missing (PR = 1.32; 95% CI 1.18-1.49), and having received prenatal care in the public sector (PR = 2.74; 95% CI 2.18-3.43) were associated with higher rates of inadequate prenatal care use. In contrast, having had prenatal care under the Family Health Program (PR = 0.92; 95% CI 0.85-0.98) was associated with a lower inadequacy rate (Table 4).

In the block of factors related to health needs, having had hypertension before or during pregnancy (PR = 0.89; 95% CI 0.80-0.98) or having had previous pregnancy losses (PR = 0.87; 95% CI 0.80-0.95) were associated with lower rates of inadequate prenatal care use. Higher rates of inadequate prenatal care use were observed for women who smoked (PR = 1.28; 95% CI 1.13-1.45) or used alcohol (PR = 1.15; 95% CI 1.05-1.26) during pregnancy (Table 4).

## Discussion

Important advances in prenatal care use have occurred in São Luís. These improvements were noted when data from the birth cohort started in 1997/98 [4, 8] was compared to the BRISA cohort data, started 13 years later. The percentage of women who did not receive prenatal care was reduced from 8.2% to 2.0%. The rate of adequate prenatal care use increased from 47.3% to 58.2%, the percentage of women who attended six or more visits increased from 34.8% to 60.5%, and the percentage of an early beginning of prenatal care during the first trimester of pregnancy rose from 56.5% to 66.2%. However, despite improved rate of adequate prenatal care use, social inequity persisted, with both low maternal schooling and low family income continuing to be associated with higher rates of inadequate prenatal care use in the later cohort. In addition, primiparae, women living in consensual union or without a companion, women who were not head of the family or who were covered by the public sector also showed higher rates of inadequate prenatal care use in both cohorts [4].

Racial disparities in the use of prenatal services were detected in the 2010 BRISA cohort, with black and mulatto women showing higher rates of inadequate prenatal care use. Adolescents, smokers or women who consumed alcohol during pregnancy also showed higher rates of inadequacy. In contrast, women with hypertension, previous pregnancy loss or those covered by the Family Health Program showed lower rates of inadequate prenatal care use.

### Universal prenatal care coverage

Several studies have shown improved care during pregnancy and delivery throughout Brazil [1, 29]. Receiving prenatal care and mainly starting the visits in the first trimester of pregnancy are a conditioning factor for a favorable outcome of gestation [30]. This early beginning guarantees time for timely implementation of health interventions such as prevention of preterm birth and of child’s and mother’s death, events not rare during pregnancy, at least in developing countries. This aspect is considered essential to ensure the quality of prenatal care [1, 25, 31]. In the present study, failure to receive prenatal care in São Luís was reduced from 8.2% in 1997/98 [4] to 2.0% in 2010. There was an increase in the percentage of completion of six or more prenatal visits, of early beginning of prenatal care in the first trimester of pregnancy and of visits provided by the public health system. Attending at least one prenatal visit is practically universal in the municipality of São Luís. However, the rate of inadequate prenatal care use is still high.

Despite these improvements, 29.8% of the women studied started prenatal care with a delay, although in 2010 the rate of appropriate use of prenatal care (58.2%) was higher than in 1997/98 (47.3%) [4]. Although practically all women attended at least one prenatal visit, it is still a challenge to provide prenatal care according to the model proposed by the Brazilian Health Ministry, which recommends that all women attend the first visit by the 12^th^ week of pregnancy and complete at least six visits [24, 32].

The rate of adequate prenatal care use considered in the present study was based on the guidelines of the Brazilian Ministry of Health [32]. This rate was used because the ones proposed by Kessner and by Kotelchuck follow the recommendations of the ACOG, which requires a large number of visits in order to consider prenatal care use to be adequate. The World Health Organization and some studies [3, 8, 12, 33] have questioned whether this number of visits may be excessive, since it has been demonstrated that there is no difference in perinatal outcomes when the number of visits of low-risk pregnant women is reduced. However, a Cochrane systematic review on the evidence for the number of prenatal visits and perinatal outcomes concluded that reduced visits programmes of antenatal care are associated with an increase in perinatal mortality compared to standard care [34].

It is difficult to compare the rate of inadequate prenatal care use in São Luís with those reported in various other studies because different classifications have been employed. Heaman *et al.* [35] showed that the Kessner and Kotelchuck indices revealed different patterns of use and resulted in various degrees of association of inadequate prenatal care with adverse pregnancy outcomes. The selection of an index of prenatal care use for program evaluation requires a careful analysis of methodological bases and limitations of the chosen index [35, 36].

A reduced rate of no prenatal care use in São Luís was noted, an aspect also observed in the state of Maranhão, of which São Luís is the capital city [23, 24]. The percentage was slightly higher than that observed in Aracaju, Sergipe, Brazil in 2005 [18] (1.7%), but lower than that observed in Corinto, Minas Gerais, Brazil, in 2003/2004 [31] (3,4%) and in Pelotas, Rio Grande do Sul, Brazil, in 2004 [37] (2.2%). In 2002, prenatal care coverage in poor areas of the North and Northeast regions of Brazil was 86% and increased to 89.8% in 2005 [1], values lower than that observed in the present study.

### Factors predisposing to the use of prenatal care services

The rates of inadequate prenatal care use decreased with increasing maternal schooling. Low maternal schooling, up to four years of study, was strongly associated with inadequate prenatal care both in 1997/98 (PR = 3.01) [4] and in 2010 (PR = 2.72). Although the educational level has considerably increased in Brazil, especially over the last few years [1, 2], this improvement did not result in reduction of inequity in adequate prenatal care use, as also observed in other studies [8, 18, 19, 21]. The persistent association of low schooling with inadequate prenatal care use shows that more socially vulnerable groups receive deficient prenatal care, demonstrating the occurrence of the “inverse health care law” according to which the individuals who need health care the most are those who have least access to it [8, 19].

The association of black and brown skin color with higher rates of inadequate prenatal care use was studied only in 2010. A study conducted in Pelotas, Rio Grande do Sul, Brazil [37] demonstrated that black women started prenatal care later and attended fewer prenatal care visits than white women. Leal *et al*. [9] reported that mulatto or black women completed fewer prenatal visits than white women. Women with low family income and black skin color and their newborns showed poorer outcomes than the remainder of the population [38], a fact possibly reflecting the presence of other social inequalities and environmental and nutritional disadvantages in addition to unequal prenatal care use. It is important to point out that racial inequity in the use of prenatal care persisted even after controlling for socioeconomic factors, suggesting that racial discrimination plays a negative role in the access to and use of prenatal care.

A reduction of the percentage of women younger than 20 years among all births was observed. Over the last few years, the reproductive profile of Brazilian women has been changing. As demonstrated in the latest survey of the Brazilian Institute of Geography and Statistics (IBGE, in the Portuguese acronym) [39], women are increasingly delaying the birth of their first child. Among the women having given birth studied here, there was an increase in the age range of 20 to 34 years and ≥35 years. Although the percentage of teenage pregnancy (<20 years) decreased in 2010, a significant value of 18.7% still persisted. Age ≥35 years became a protective factor against inadequate prenatal care use in 2010. Conversely, adolescents had a greater chance of inadequate prenatal care use, as also observed in the Brazilian cities of Palmas in 2009 [19] and Aracaju in 2005 [18].

Being a primipara was a protective factor against inadequate prenatal care use, whereas multiparous women had more chances of receiving inadequate prenatal care. This result was also obtained in 2002 by Trevisan *et al.* [21]. These authors raised two possible hypotheses to explain these associations: the larger the number of children a woman has, the more she will consider herself self-sufficient in dealing with future generations, or, conversely, prenatal care received in previous pregnancies did not convince her of its importance. According to PNDS-2006 (Demographic and Health Survey in the Portuguese acronym) data, access to prenatal care tends to decrease with increasing number of children [40, 41].

Another important finding of the present study is that, when the mother is not the head of the family, rates of inadequate prenatal care use are higher, even after adjustment for socioeconomic factors. It suggests that, when women have greater autonomy for making decisions about family matters, health outcomes are better.

### Factors facilitating the use of prenatal care – enabling resources

Income inequalities, although still strongly present in Brazil, have shown a decreasing trend [1]. The recent expansion of cash transfer programs focused on the poorest population, such as the “Bolsa Família” (Family Allowance) and the Benefit of Continued Provision of Social Assistance, among others, are contributing to internal redistribution among the various parts of the total family income [39]. Despite reducing socioeconomic inequalities, in the present investigation we still observed persistent social inequity in the adequate use of prenatal care since poorer and less educated women had higher rates of inadequate prenatal care use.

In the present study, public prenatal care was strongly associated with inadequate prenatal care use, a result similar to those observed in various studies [4, 42, 43] that compared prenatal care coverage between public and private sectors, showing a sharp advantage for pregnant women attended in the private sector. Important differences in the quality of prenatal care between public and private institutions were also observed by Victora *et al.* in Pelotas [37]. However, when comparing inadequacy of prenatal care use in the earlier cohort, 1997/98, with present data, it is worth noting that improvements in the adequacy of prenatal care use were only observed in the public sector. Inadequacy of prenatal care use was reduced from 53% in 1997/98 [4] to 43.6% in 2010 (p < 0.001) in this sector, whereas in the private sector this rate did not change from 1997/98, 6.2% [4], to 2010, 10,1% (P = 0.058). As a consequence, differences in prenatal care use between the public and the private sector were reduced (prevalence ratio nearly halved from 8.47 in 1997/98 [4] to 4.34 in 2010). This may be due to the fact that prenatal care provided by the private sector expanded from 11.6% in 1997/98 [4] to 18.3% in 2010 and at the same time more companies included health insurance as a benefit for their manual workers in 2010 than they did in 1997/98. Since manual workers are poorer they tend to attend fewer prenatal care visits [17]. Alternatively another explanation could be the “inverse equity hypothesis”. Since the rich achieved a very low level of inadequacy of prenatal care use, the poor gained greater access to interventions and this phenomenon tends to reduce inequity [44].

Prenatal care coverage by the Family Health Program was associated with a lower rate of inadequate prenatal care use. This result was similar to what has been observed in studies conducted in Rio Grande do Sul, Brazil [43, 45], where the percentages of adequate prenatal care provided by the Family Health Program were higher than those observed in the traditional health facilities, closely followed by those for women attending health-plan clinics and private offices. This difference may be attributed to the work process in the FHP, which includes a community health worker that actively searches pregnant women and refers them to early initiation of prenatal care. In addition, since care is centered on the family, this leads to bond formation between the professionals and the women. Furthermore, a multiprofessional team of a physician, a nurse and a CHW is involved, and care is organized by geographical area [46].

These data demonstrate that, in Brazil, the FHP has contributed to the reduction of inequities in the use of health services and has favored equality. It should be pointed out that analyses at the municipal level have clearly shown that the FHP had a positive effect on the reduction of child mortality in different regions of the country [47, 48]. This reduction may be attributed to increased coverage of prenatal care and to adequate management of common childhood diseases, both promoted by the FHP [27].

### Need factors for the use of prenatal care

Having had hypertension before or during pregnancy (PR = 0.89; 95% CI 0.80-0.98) was associated with a lower rate of inadequate prenatal care use. This finding is different from that observed in Rio de Janeiro [49], where the rate of adequate prenatal care use did not differ between pregnant women with arterial hypertension and pregnant women considered to be at low risk.

However, the same study [49] called attention to the fact that arterial hypertension per se may not be a sufficient factor for a pregnant woman not to receive adequate prenatal care. The professionals provide greater care when a woman reports a history of an unfavorable outcome of a previous pregnancy, indicating that they are more committed to repairing an unsuccessful event than to preventing a future one. The pregnant women with an unfavorable obstetrical history were probably also more concerned about taking care of themselves, using medication and seeing a specialist, thus contributing to better results of prenatal management. This result agrees with that observed in the present study, in which a previous pregnancy loss was associated with a lower rate of inadequate prenatal care use. However, a history of preterm birth was not associated with a higher rate of inadequate prenatal care use.

Other studies [10, 18, 50, 51] have demonstrated an association of maternal smoking habit and alcohol intake with inadequate utilization of prenatal care, as also observed in the present study. According to Vigitel data [38], the prevalence of smoking in Brazil fell from 35% in 1989 to 16% in 2006. The prevalence of the smoking habit remained stable for women, being 12.7% in 2006 and 12.5% in 2009 [39]. The Brazilian prevalence rates are lower than those detected in neighboring countries, possibly as the result of a smoking control policy implemented in the country since the 1990’s. The quality and impact of the National Program for Smoking Control have been highly recognized at the international level [42]. Exposure to alcohol also affects the health of the mother, causing cardiovascular diseases, cancer, depression, and neurological disorders. In addition, it is associated with insufficient gestational weight gain and an increased risk of using other drugs [51].

### Strengths

One of the advantages of the present study was the use of an index that seems to be more appropriate for the Brazilian reality, as recommended by the Ministry of Health. This index also took into account gestational age in order to consider a given number of visits to be adequate, a fact that minimizes the bias of erroneously attributing lower adequacy of prenatal care use to preterm pregnancies. Another advantage was the comparison to the study conducted in São Luís in 1997/98 [4], which used the same criteria for the classification of adequate prenatal care use, allowing us to compare the percentage of prenatal care utilization in São Luís at different times. In addition, the two studies randomly sampled at least 94% of all births that occurred during the two study periods, a fact that reinforces generalization of the results.

### Limitations

At least one limitation of the study concerned the methods used to determine the time when prenatal care was started and the number of prenatal care visits in order to assess the adequacy of prenatal care use. The indices of adequate use of prenatal care are based on the number of visits recommended for a low-risk pregnancy, but they do not establish any recommendation of a standard number of visits for women at high risk and do not contemplate the intervals between visits.

Another limitation is the use of self-reported data. In addition, it was not possible to assess the efficacy or quality of the visits since the indices are quantitative, this being a limitation observed in most studies [7, 8, 52, 53]. Since 2% of births were home births, it is likely that women who do not deliver in hospitals have different prenatal behaviours compared to those that deliver in hospitals. However, even with these limitations, the indices for the evaluation of adequate use of available prenatal care provide useful information.

## Conclusions

Prenatal care coverage was practically universal and there was a reduction in the rate of its inadequate utilization. Both predisposing and enabling factors, as well as factors related to health needs were associated with inadequate utilization of prenatal care. However, social and racial disparities still persist in the use of this care, even after the improvement of income and schooling observed during this period. Being poor, black or mulatto and having a low educational level are important barriers against receiving adequate prenatal care. The present data support the importance of the Family Health Program as a model of organization of health care, since women covered by this program had lower rates of inadequate utilization of prenatal care.
